# Supplementary material for: Glycolytic disruption restricts Drosophila melanogaster larval growth via the cytokine Upd3
Source: PLoS Genet. 2025 May 2;21(5):e1011690. doi: 10.1371/journal.pgen.1011690 (PMC12068724; doi:10.1371/journal.pgen.1011690)
Supplement: S1 Fig — Representative confocal images of L2 larval tissues expressing Ldh-GFPGenomic and immuno-stained with αGPDH1 antibody. DAPI is shown in blue, Ldh-GFP and Gpdh1 are represented in green and magenta, respectively. The rightmost panel displays the merged images of Ldh-GFP and Gpdh1 staining. (A-D) Intestine; (E-H) Dorsal side of CNS; (I-L) fat body; (M-P) salivary gland. Scale bars in leftmost panels represents 40 μM and applies to all other panels in the same row. (PDF) [file pgen.1011690.s001.pdf]

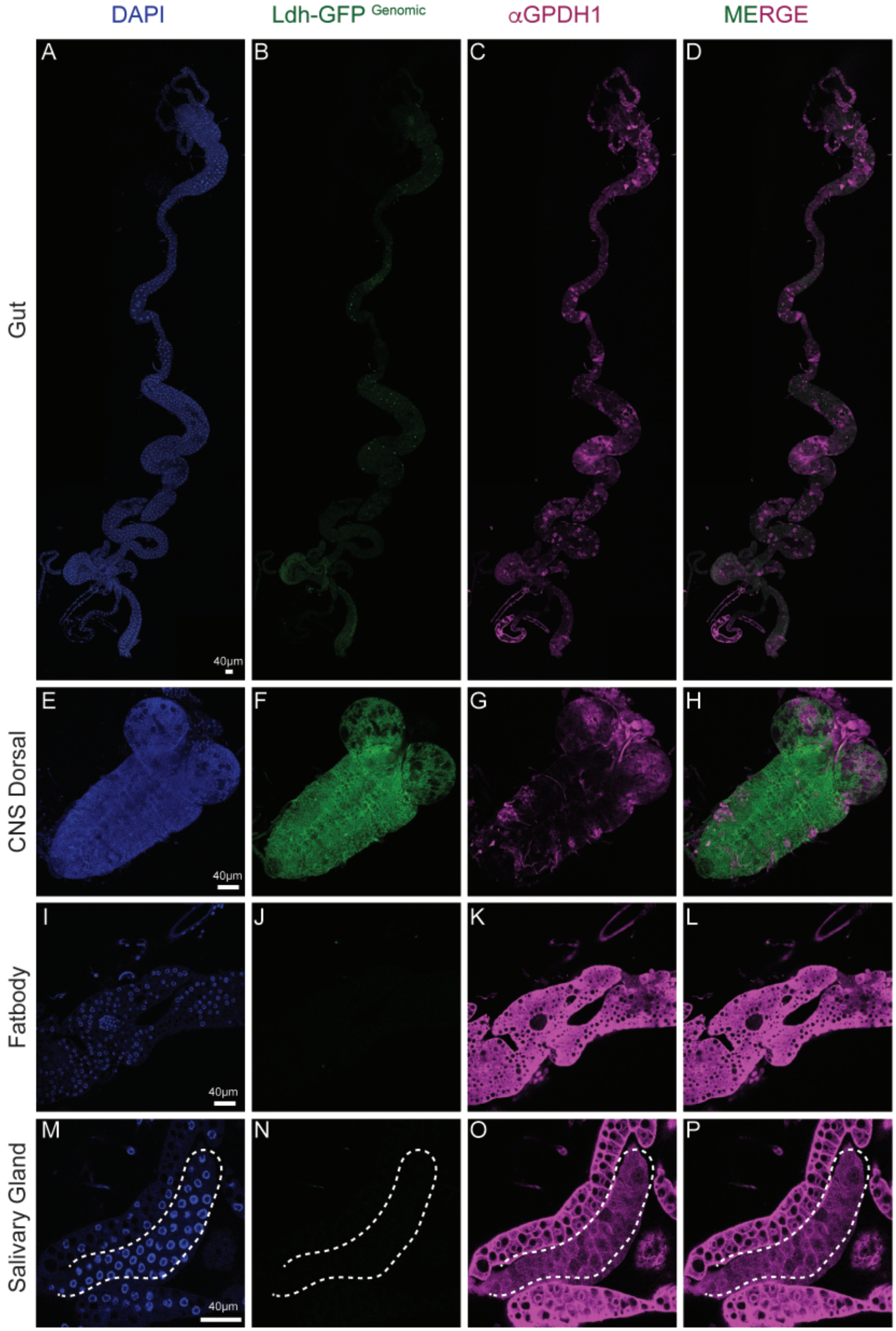

S1 Fig. Ldh and Gpdh1 expression patterns in the CNS, fat body, salivary gland, and intestine. Representative confocal images of L2 larval tissues expressing *Ldh-GFP^{Genomic}* and immuno-stained with αGPDH1 antibody. DAPI is shown in blue, Ldh-GFP and Gpdh1 are represented in green and magenta, respectively. The rightmost panel displays the merged images of Ldh-GFP and Gpdh1 staining. (A-D) Intestine; (E-H) Dorsal side of CNS; (I-L) fat body; (M-P) salivary gland. Scale bars in leftmost panels represents 40 µM and applies to all other panels in the same row.
