# Supplementary material for: Glycolytic disruption restricts Drosophila melanogaster larval growth via the cytokine Upd3
Source: PLoS Genet. 2025 May 2;21(5):e1011690. doi: 10.1371/journal.pgen.1011690 (PMC12068724; doi:10.1371/journal.pgen.1011690)
Supplement: S3 Fig — Quantification of Ldh and Gpdh1 expression in the larval fat body of the heterozygous control strain (Gpdh1A10/+; Ldh16/+) and each single mutant strain (Gpdh1A10/B18 and Ldh16/17). (A-F) Representative confocal images of (A-C) Ldh expression and (D-F) DAPI staining in all three genotypes. (G) Ldh staining was quantified in the fat body of all three genotypes. (H-M) Representative confocal images of (H-J) Gpdh1 expression and (K-M) DAPI staining in all three genotypes. (N) Gpdh1 staining was quantified in the larval fat body of all three genotypes. The scale bar in all images represents 40 μM. The scale bar in (A) applies to (B-F) and the scale bar in (H) applies to (I-M). (G, N) Data presented as a scatter plot with the lines representing the mean and standard deviation. P-values were calculated using an ANOVA followed by a Holm-Sidak test. ***P < 0.01. (PDF) [file pgen.1011690.s003.pdf]

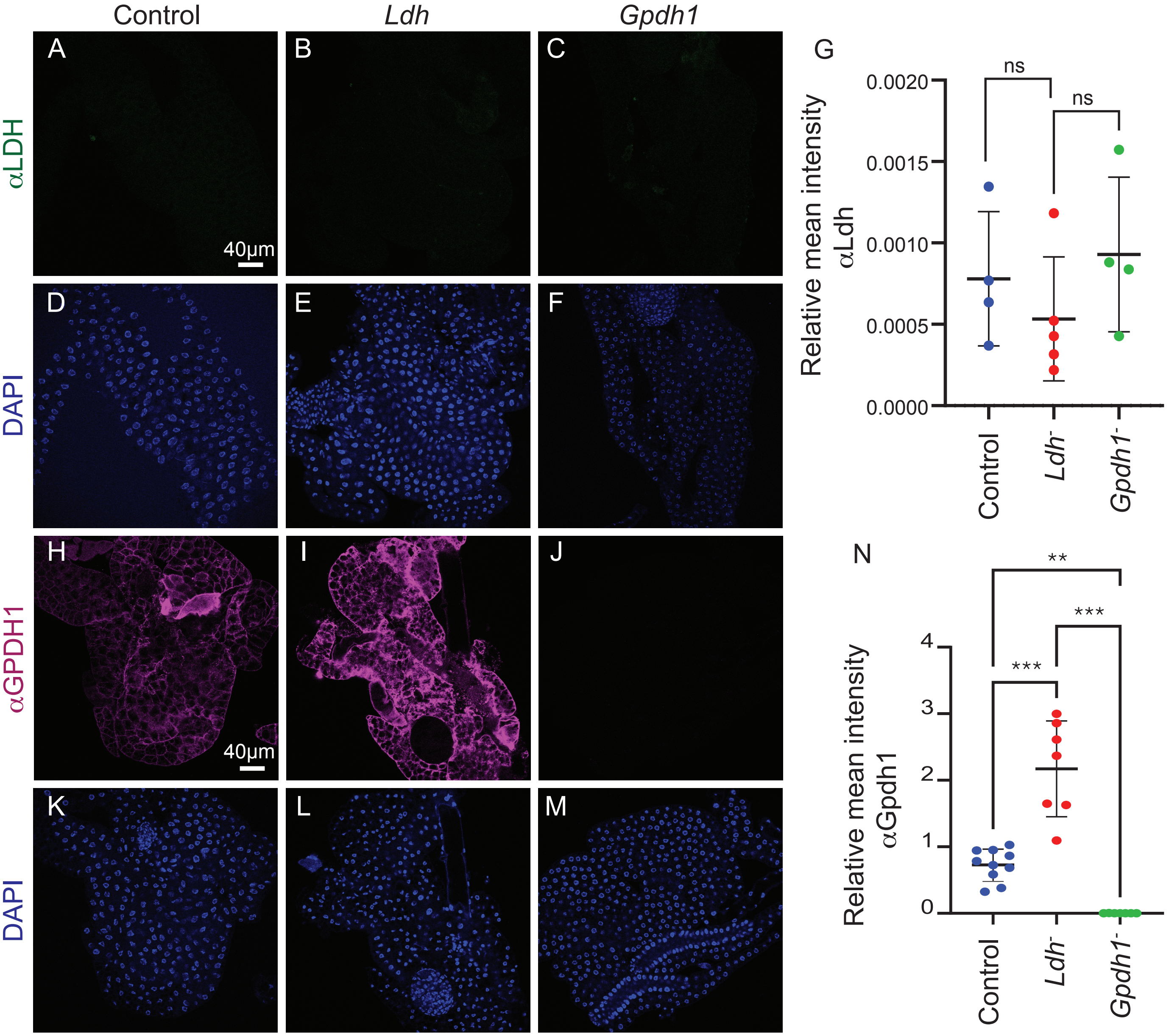

S3 Fig. Characterization of either Ldh or Gpdh1 expression in larval fat body following loss of the other enzyme. Quantification of Ldh and Gpdh1 expression in the larval fat body of the heterozygous control strain ($Gpdh1^{A10/+}$; $Ldh^{16/+}$) and each single mutant strain ($Gpdh1^{A10/B18}$ and $Ldh^{16/17}$). (A-F) Representative confocal images of (A-C) Ldh expression and (D-F) DAPI staining in all three genotypes. (G) Ldh staining was quantified in the fat body of all three genotypes. (H-M) Representative confocal images of (H-J) Gpdh1 expression and (K-M) DAPI staining in all three genotypes. (N) Gpdh1 staining was quantified in the larval fat body of all three genotypes. The scale bar in all images represents 40 µM. The scale bar in (A) applies to (B-F) and the scale bar in (H) applies to (I-M). (G, N) Data presented as a scatter plot with the lines representing the mean and standard deviation. *P*-values were calculated using an ANOVA followed by a Holm-Sidak test. ****P* < 0.01.
