# Supplementary material for: Glycolytic disruption restricts Drosophila melanogaster larval growth via the cytokine Upd3
Source: PLoS Genet. 2025 May 2;21(5):e1011690. doi: 10.1371/journal.pgen.1011690 (PMC12068724; doi:10.1371/journal.pgen.1011690)
Supplement: S4 Fig — Representative confocal images of mCherry expression in (A-B) larval muscles, (C-D) gut muscles, (E-F) CNS, (G-H) fat body, and (I-J) salivary gland at 74–80 hrs after egg-laying. DAPI is shown in blue and Mef2R-Gal4 driven 20XUAS-6XmCherry expression in red. The scale bars represent 50 μM. (PDF) [file pgen.1011690.s004.pdf]

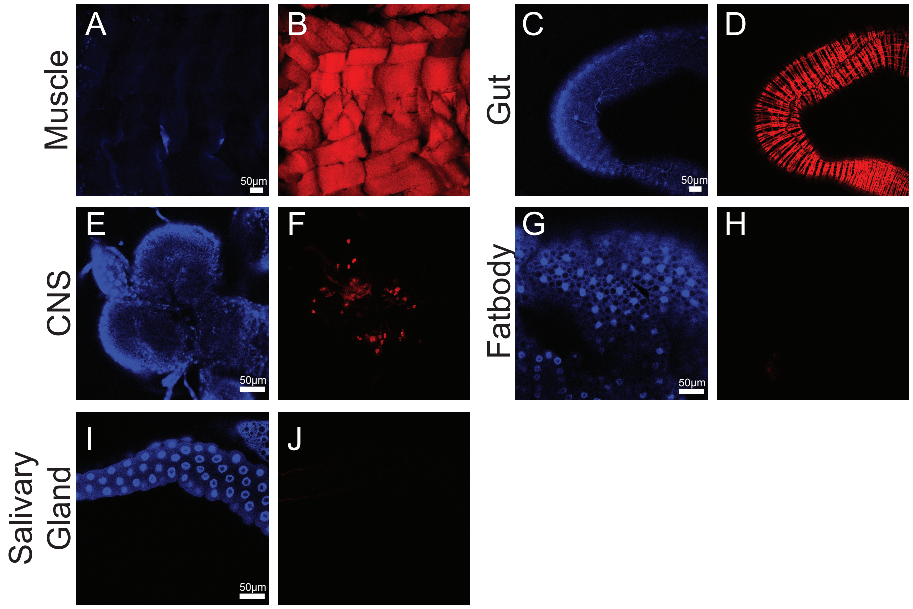

S4 Fig. *Mef2R-Gal4* driven expression of 20XUAS-6XmCherry in larval tissues. Representative confocal images of mCherry expression in (A-B) larval muscles, (C-D) gut muscles, (E-F) CNS, (G-H) fat body, and (I-J) salivary gland at 74-80 hrs after egg-laying. DAPI is shown in blue and *Mef2R-Gal4* driven 20XUAS-6XmCherry expression in red. The scale bars represent 50 µM.
