# Supplementary material for: Glycolytic disruption restricts Drosophila melanogaster larval growth via the cytokine Upd3
Source: PLoS Genet. 2025 May 2;21(5):e1011690. doi: 10.1371/journal.pgen.1011690 (PMC12068724; doi:10.1371/journal.pgen.1011690)
Supplement: S5 Fig — Representative confocal images of muscles 74–80 hrs after egg-laying from (A) control, (B) Gpdh1-, (C) Mef2R-Gal4 driven UAS-Gpdh1-RNAi stained with anti-Gpdh1 antibody. The scale bars represent 50 μM. (D) Quantification of the mean intensity of Gpdh1 in all conditions. Data presented as a scatter plot with the lines representing the mean and standard deviation. P-values were calculated using an ANOVA followed by a Holm-Sidak test. *P < 0.05. (PDF) [file pgen.1011690.s005.pdf]

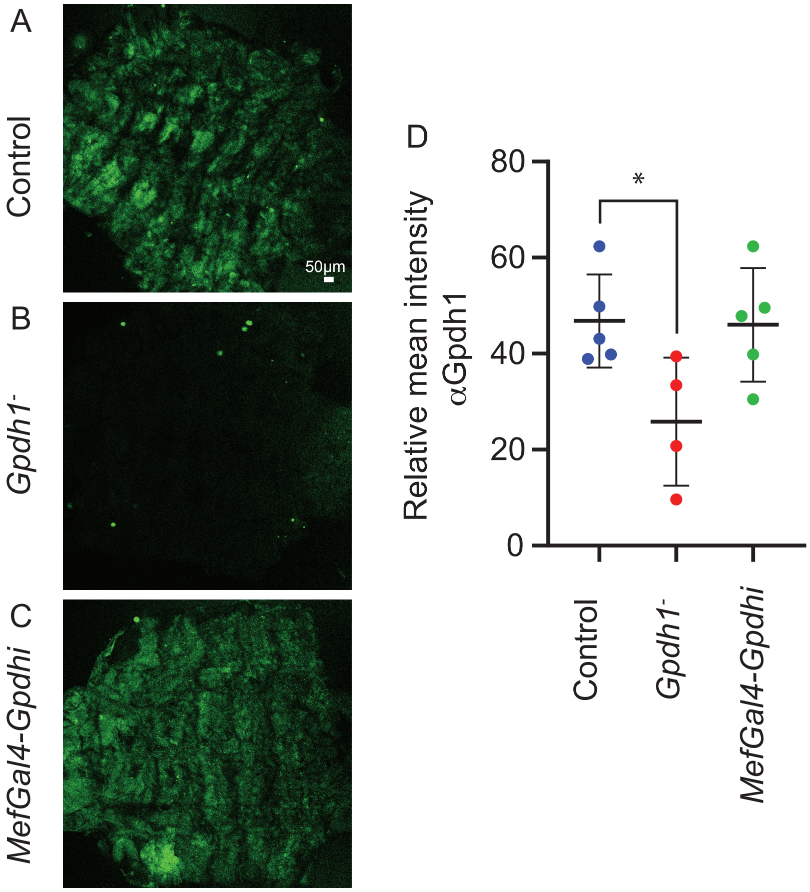

S5 Fig. The transgene to knockdown *Gpdh1* does not significantly reduce Gpdh1 levels in the muscles. Representative confocal images of muscles 74-80 hrs after egg-laying from (A) control, (B) *Gpdh1*$^{-}$, (C) *Mef2R-Gal4* driven *UAS-Gpdh1-RNAi* stained with *anti-Gpdh1* antibody. The scale bars represent 50 µM. (D) Quantification of the mean intensity of Gpdh1 in all conditions. Data presented as a scatter plot with the lines representing the mean and standard deviation. *P*-values were calculated using an ANOVA followed by a Holm-Sidak test. *$P < 0.05$.
