# Supplementary material for: Glycolytic disruption restricts Drosophila melanogaster larval growth via the cytokine Upd3
Source: PLoS Genet. 2025 May 2;21(5):e1011690. doi: 10.1371/journal.pgen.1011690 (PMC12068724; doi:10.1371/journal.pgen.1011690)
Supplement: S6 Fig — Volcano plot depicting the transcriptomic profiles of (A) Ldh mutants (Ldh16/17), (B) Gpdh1 mutants (Gpdh1A10/B18), and (C) double mutants (Gpdh1A10/B18; Ldh16/17) relative to the respective heterozygous control strains. n = 3 biological replicates analyzed per genotype. Each sample contained 20 mid-L2 larvae. Vertical axis indicates -log10(FDR) and horizontal axis represents log (FC). The significantly upregulated genes are shown in yellow and downregulated are shown in black. FDR- fold discovery rate and FC-fold change. (PDF) [file pgen.1011690.s006.pdf]

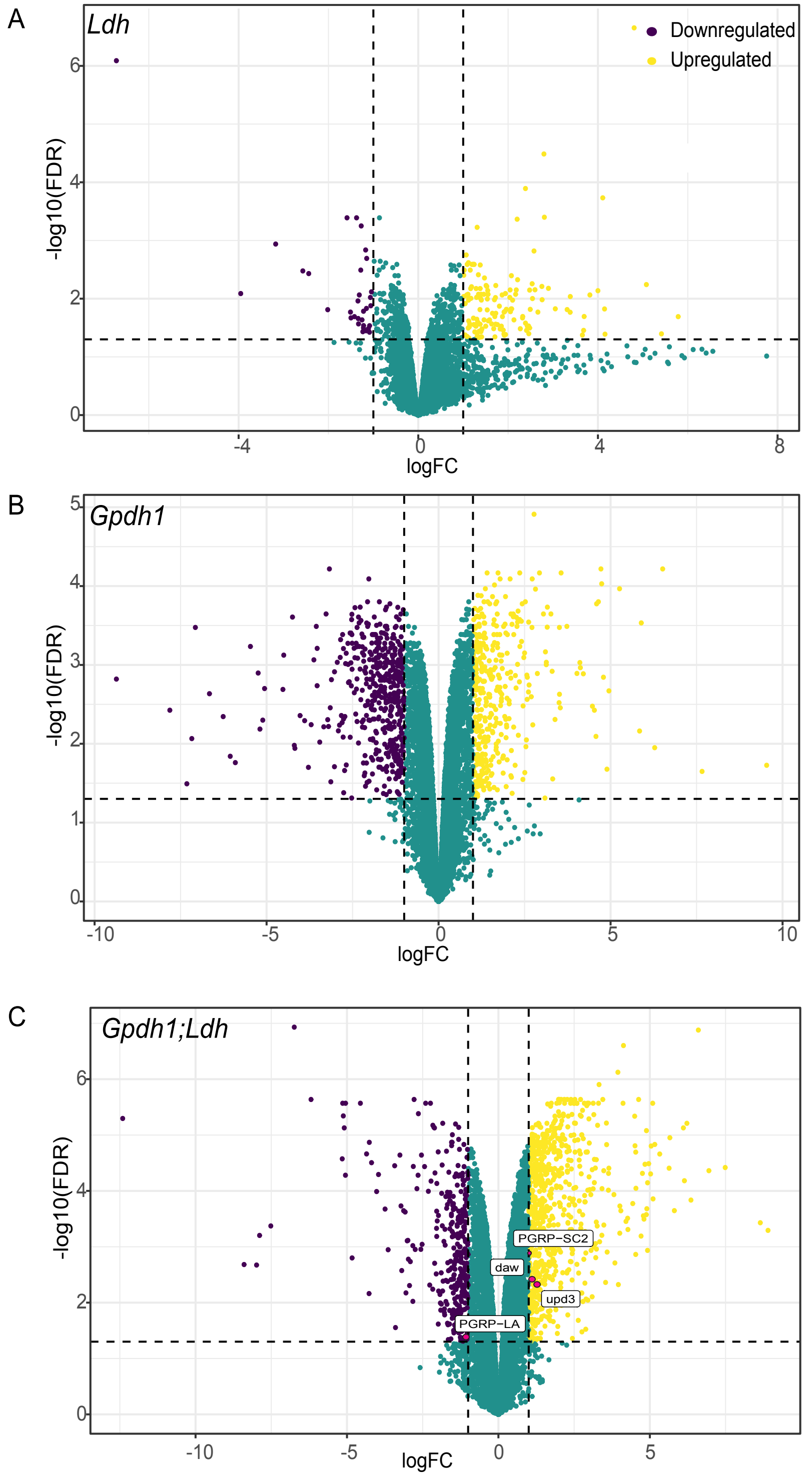

S6 Fig. RNA-seq analysis of *Ldh* mutants, *Gpdh1* mutants, and *Gpdh1, Ldh* double mutants. Volcano plot depicting the transcriptomic profiles of (A) *Ldh* mutants ($Ldh^{16/17}$), (B) *Gpdh1* mutants ($Gpdh1^{A10/B18}$), and (C) double mutants ($Gpdh1^{A10/B18}$; $Ldh^{16/17}$) relative to the respective heterozygous control strains. n=3 biological replicates analyzed per genotype. Each sample contained 20 mid-L2 larvae. Vertical axis indicates -log10(FDR) and horizontal axis represents log (FC). The significantly upregulated genes are shown in yellow and downregulated are shown in black. FDR- fold discovery rate and FC-fold change.
