# Supplementary material for: Glycolytic disruption restricts Drosophila melanogaster larval growth via the cytokine Upd3
Source: PLoS Genet. 2025 May 2;21(5):e1011690. doi: 10.1371/journal.pgen.1011690 (PMC12068724; doi:10.1371/journal.pgen.1011690)
Supplement: S7 Fig — Samples represented in Fig 4 were quantified for pJNK staining intensity. Data presented as a scatter plot with the lines representing the mean and standard deviation. P-values were calculated using an ANOVA followed by a Holm-Sidak test. ***P < 0.001. (PDF) [file pgen.1011690.s007.pdf]

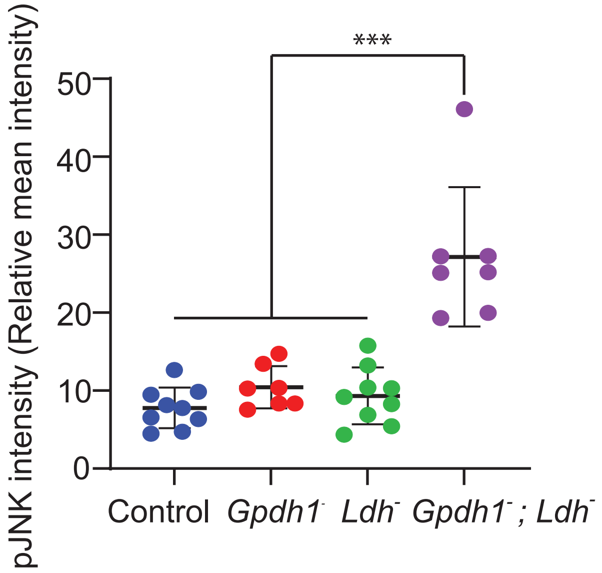

S7 Fig. Quantification of pJNK intensity in the muscles. Samples represented in Fig 4 were quantified for pJNK staining intensity. Data presented as a scatter plot with the lines representing the mean and standard deviation. *P*-values were calculated using an ANOVA followed by a Holm-Sidak test. ****P*<0.001.
