# Supplementary material for: Glycolytic disruption restricts Drosophila melanogaster larval growth via the cytokine Upd3
Source: PLoS Genet. 2025 May 2;21(5):e1011690. doi: 10.1371/journal.pgen.1011690 (PMC12068724; doi:10.1371/journal.pgen.1011690)
Supplement: S9 Fig — (A-L) Representative confocal images of (A-D) fat body, (E-H) salivary glands and (I-L) muscles showing Stat-GFP expression in control, Gpdh1A10/B18, Ldh16/17 and Gpdh1A10/B18; Ldh16/17 double mutants at 74–80 hrs AEL. The scale bar represents 40 μM. The scale bar in (A) applies to (B-L). (M-O) Quantification of the relative mean intensity (RMI) of Stat-GFP in fat body (M), salivary glands (N) and muscles (O). Data presented as a scatter plot with the lines representing the mean and standard deviation. P-values were calculated using an ANOVA followed by a Holm-Sidak test. **P < 0.01. (PDF) [file pgen.1011690.s009.pdf]

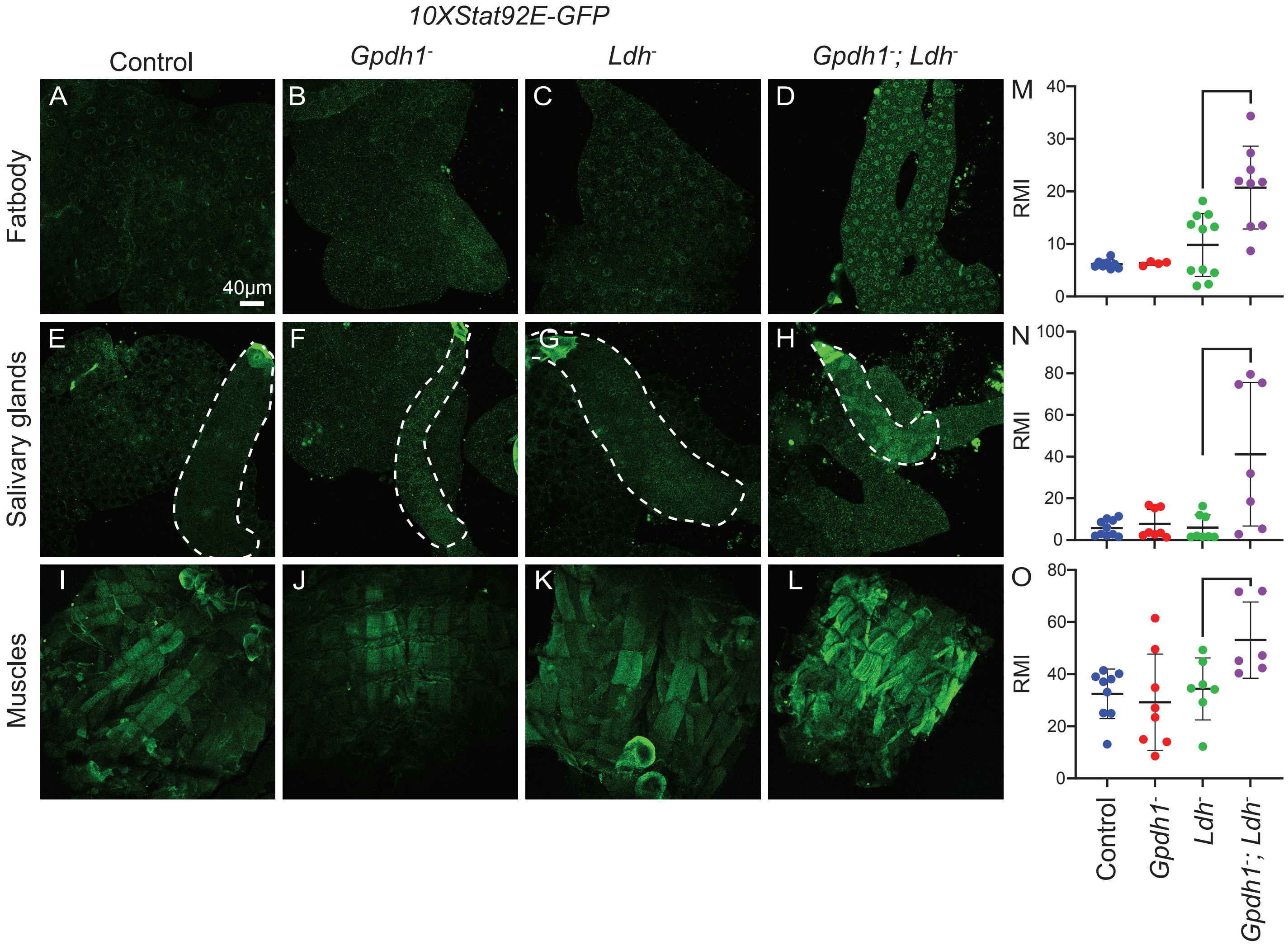

S9 Fig. *Stat-GFP* expression is increased in larval tissues of *Gpdh1; Ldh* double mutants. (A-L) Representative confocal images of (A-D) fat body, (E-H) salivary glands and (I-L) muscles showing *Stat-GFP* expression in control, $Gpdh1^{A10/B18}$, $Ldh^{16/17}$ and $Gpdh1^{A10/B18}$; $Ldh^{16/17}$ double mutants at 74-80 hrs AEL. The scale bar represents 40 μM. The scale bar in (A) applies to (B-L). (M-O) Quantification of the relative mean intensity (RMI) of *Stat-GFP* in fat body (M), salivary glands (N) and muscles (O). Data presented as a scatter plot with the lines representing the mean and standard deviation. *P*-values were calculated using an ANOVA followed by a Holm-Sidak test. $**P < 0.01$.
