# Supplementary material for: Glycolytic disruption restricts Drosophila melanogaster larval growth via the cytokine Upd3
Source: PLoS Genet. 2025 May 2;21(5):e1011690. doi: 10.1371/journal.pgen.1011690 (PMC12068724; doi:10.1371/journal.pgen.1011690)
Supplement: S10 Fig — (A) A graph showing the increased level of 20E in upd3Δ mutants at the mid-L2 stage (60–66 hrs after egg-laying). (B) A graph illustrating the percent of Gpdh1A10/B18 and Ldh16/17 single mutants that pupated when raised on yeast-molasses agar that contains either ecdysone or the solvent (ethanol) control. All experiments are repeated a minimum of three times. n = 9 biological replicates. Data presented as a scatter plot with the lines representing the mean and standard deviation. P-values were calculated using an ANOVA followed by a Holm-Sidak test. *P < 0.05. (PDF) [file pgen.1011690.s010.pdf]

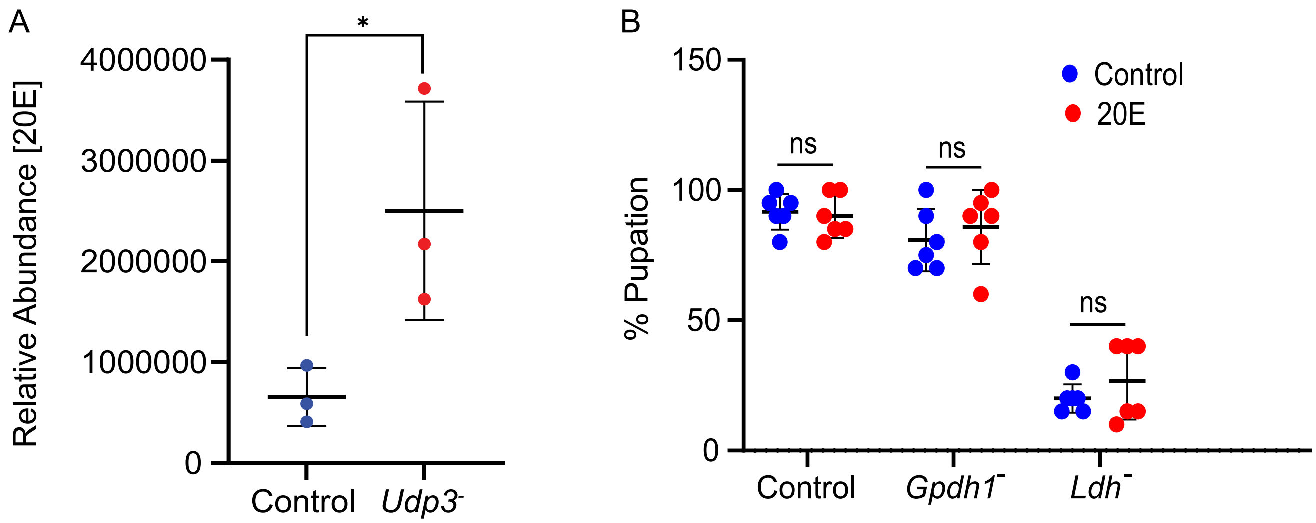

S10 Fig. Loss of Upd3 increases 20E and dietary supplementation with 20E has no effect on *Gpdh1* and *Ldh* single mutant larval growth. (A) A graph showing the increased level of 20E in *upd3*$^{\Delta}$ mutants at the mid-L2 stage (60-66 hrs after egg-laying). (B) A graph illustrating the percent of *Gpdh1*$^{A10/B18}$ and *Ldh*$^{16/17}$ single mutants that pupated when raised on yeast-molasses agar that contains either ecdysone or the solvent (ethanol) control. All experiments are repeated a minimum of three times. n=9 biological replicates. Data presented as a scatter plot with the lines representing the mean and standard deviation. *P*-values were calculated using an ANOVA followed by a Holm-Sidak test. *$P < 0.05$.
