# Supplementary material for: Glycolytic disruption restricts Drosophila melanogaster larval growth via the cytokine Upd3
Source: PLoS Genet. 2025 May 2;21(5):e1011690. doi: 10.1371/journal.pgen.1011690 (PMC12068724; doi:10.1371/journal.pgen.1011690)
Supplement: Attachment — Submitted filename: Rai_Response to Reviewer Comments.docx [file pgen.1011690.s016.docx]

**Response to Reviewers Comments and Questions**

We thank the Reviewer’s for their helpful advice. Below we respond to comments, concerns and suggestions. In response to the Reviewer’s requests, we have added a substantial amount of new data that significantly improves the manuscript.

**Reviewer #1:** The study stems from the intriguing finding that disrupting glycolytic activity in the larval muscle, does not only affect growth locally, but induces a systemic growth defect and developmental arrest. Following this observation, the authors finds that the developmental arrest induced in the Gpdh1; Ldh double mutant animals leads to increased expression of a cytokine Upd3 and elevated Jak/Stat receptor activity in the prothoracic gland of the CNS. Furthermore, the authors show that loss of Upd3, as well as exogenous ecdysone administration, can rescue the developmental defects caused by Gpdh1; Ldh double mutant. Based on these findings, the authors suggest a model where disruption of glycolytic activity in the muscle, result into cellular stress and growth defect, which is then coordinated at the systemic level through Upd3 and ecdysone signaling.

While the mechanism, Upd3 signal to prothoracic gland, has been already shown elsewhere (https://doi.org/10.1016/j.cub.2021.01.080), the study of Rai et al. demonstrate its utilization in an unexpected context. Indeed, Rai et al. tackles important, and still poorly understood, questions of how central metabolism is interconnected with cellular signaling pathways, and how animal growth is physiologically coordinated in general. Although many underlying mechanical details are left unanswered, the work of Rai et al. significantly improves our understanding of these topical questions. Overall, the manuscript is very well written, the methods and the data are clearly presented, and the data is supporting the conclusions. Therefore, I find this study to be important, and interesting to the readers of PLOS Genetics. However, to make some of the key findings more convincing, I would ask the authors to add few more control experiments to the manuscript. Please find my specific comments and suggestion figure by figure below.

Thank you for the kind words and helpful comments.

Figure 1.
Please change aGPDH to αGPDH.

We have made the change in Figure 1.

Figure 2.
The data in figure 2 are central to the study since it shows the tissue specificity of glycolytic disturbance to systemic growth. The data presented is clear, however it relies on single Ldh RNAi line. Since there is no apparent phenotype for to judge the functionality of this RNAi line, the reader is left wondering its efficiency. Thus, to make the story more convincing I kindly ask the authors to add either (1) functional confirmation of the used RNAi line, (2) the results of using an independent Ldh RNAi line, or preferentially (if feasible) (3) use of a reciprocal strategy with Ldh mutant combined with Gpdh knockdown in the muscle.

We previously demonstrated that the Ldh-RNAi line used in this study effectively reduces LDH protein levels (See Li et al. 2017), and have now noted this in the text (see lines 480-481). We’ve attempted the reciprocal experiment using *Gpdh1* RNAi, however, multiple TRiP lines have failed to effectively reduce the expression of Gpdh1 protein, as determined by antibody staining. We’ve noted this in the text and included a supplemental figure (Figure S5) describing this problem.

Figure 3.
Please mark the highlighted genes in Figure 3 to the volcano plots in Figure S5.

We have labeled the highlighted genes of Figure 3 in Figure S6.

The authors performed RNAseq experiment to reveal transcriptional changes in Gpdh1; Ldh double mutant larvae. They identify the cytokine Upd3 as a possible candidate for regulating the systemic growth effect. Since the RNAseq experiments are performed from whole animals, it is left unanswered if Upd3 expression is indeed affected in the muscle. This is important especially in the light of the modest change in Upd3 expression (LogFC 1.3). To make their conclusion solid, the authors should provide some evidence that Upd3 expression is affected specifically in the larval muscle in the Gpdh1; Ldh double mutant animals.

We agree with the reviewer on this point. In response, we analyzed *upd3* expression within the muscle using *upd3-Gal4* to drive expression of a *UAS-GFP* transgene (see Figure 3). Our studies revealed significantly elevated GFP expression in *Gpdh1; Ldh* double mutants as compared with both the single mutants and the controls.

The morphology of the double mutant muscle (panel F) seems very different from the single mutant controls. Could the authors comment this?

Thank you for highlighting this apparent difference. This was unintentional and we hadn’t noted a consistent difference in muscle morphology. In response to this comment, we repeated the experiment and again failed to find an obvious morphological defect. We have included new images in the figure.

Figure 4.
The rescue of the Gpdh1; Ldh double mutant by Upd3 mutation is remarkable, and the lack of detectable metabolic restoration supports the hypothesis that Upd3 works through a different mechanism. This is a very nice experiment indeed.

Thank you! Please note that these data are now found in Figure 6 and S8.

Please indicate in the figure panel:
A, B & D: At which time point are these measurements?

We have added the time points in Figure legends. Note that this is now Figure 6.

Figure 5.
Panels A-D: Please indicate how consistent this result is? The reader is especially interested in the PG Stat92E-GFP expression.

To show the consistency and significance of the increase in *Stat92E-GFP* expression in double mutants in comparison to controls, we have added the quantification of *Stat92E-GFP* expression for all the tissues including PG (Figure 8 and S9).

Please add a description of the “white dashed line indicating PG” in panels A-D (to the figure legend).

We have added the description of the “white dashed line indicating PG” in panels A-D to the legend for Figure 8.

The finding that the Gpdh1; Ldh double mutant developmental arrest is partially rescued by 20E feeding is striking. The reader is, however, left puzzled how specific this is to the genotype, or if extra 20E has a more general beneficial effect to larval growth. Therefore, I would suggest adding control genotypes, such as the Gpdh1 and Ldh single mutants to the panel E. In addition, please indicate if the 20E feeding led to any viable adults.

We agree that that these are important controls. In response to the reviewer’s comments, we fed 20E to both *Ldh* and *Gpdh1* single mutant larvae. Neither genotype displays any difference in percentage pupation when fed with 20E (Figure S10B).

We’ve also noted that while 20E feeding accelerates larval development, the animals die as pupae (Lines 309-310). We are uncertain as to why 20E can partially suppress the growth arrest but not restore normal pupal development – we’ve added text to the results and discussion raising possible explanations (Line 311-313).

Finally, we’ve added additional results that complement the rescue experiment. The previous study implicating Upd3 in regulation of 20E signaling did not measure 20E titers in *upd3* mutants. To further support our findings, we used LC-MS to measure 20E in *upd3* mutants. Consistent with our model, we found that 20E levels are elevated in *upd3* mutants (Figure S10A). Unfortunately, we were unable to collect sufficient quantities of synchronized *Gpdh1; Ldh* double mutants or *upd3; Gpdh1; Ldh* triple mutants to reliably measure 20E. Regardless, the elevated levels of 20E in *upd3* mutants provides an additional piece of evidence that supports our model.

Minor corrections/comments:
Line 130 “many cells the CNS” to “many cells of the CNS”

We have corrected this (Line 131).

Line 132 “high levels Gpdh1” to “high levels of Gpdh1”

We have corrected this (Line 133/134).

Line 134 Please add reference here.

We have added the reference (Line 135).

Line 228 Please remove “significantly” since no quantification is done here.

We have measured and added the quantification for *Stat92E-GFP* in the figure so that the use of the term significant is now appropriate.

Lines 255-257 The authors avoid mentioning the specific tissue, muscle, when describing their model. Is there a specific reason for that? To me, the tissue of the metabolic disturbance, and the source of Upd3, seems especially revealing. Could the authors comment on this issue, and perhaps mention it in the discussion?

We now specifically mention muscle in our model (see discussion) and have added additional data showing the functional importance of Upd3 expression within muscle (see Figures 3, 5, and 7). Of note, RNAi depletion of Upd3 specifically within muscle can partially suppress the double mutant phenotype, however, the suppression is not as strong as the upd3 mutant. One explanation for this observation is that other tissues are also producing Upd3.

Lines 322-327 The description of using tissue specific Ldh RNAi is confusing. Could the authors correct it?

We have clarified the text.

Line 460 Please omit “The scale bar in (A) applies to all panels”

We have omitted the line.

Line 574 Omit “in”

This has been corrected.

**Reviewer #2: Summary**
In this manuscript, Rai et al provide evidence that the larval arrest phenotype observed in Ldh;Gpdh1 double mutants is not likely due to effects on metabolism, but instead is regulated in part by growth factor signaling. While the manuscript is well written and data are solid, the authors should provide stronger rationale in some cases. Additionally, quantification of IF data can make some conclusions stronger. Overall, this work advances understanding of regulation of larval growth during Drosophila development.

Major concerns
Line 190-191: The focal plane for the double mutant seems to be different from that shown for the control and single mutants. Since the pJNK signal is nuclear, the control and single mutant representative images should be at a focal plane that contains more nuclei. On a related note, quantification and/or showing more than one image for each (in a supplemental figure) would strengthen this result.

We repeated the pJNK staining and now include improved images in Figure 4 and included quantification in Figure S7. The new images consist of comparable focal planes for all genotypes.

While the authors propose that the larval arrest phenotype in Ldh;Gpdh1 double mutants is due to defects in systemic growth factor signaling, they did not examine signaling pathways known to control larval growth, namely insulin/insulin-like growth factor or mTOR-mediated signaling. To provide more direct support of their hypothesis, the activity of these pathways in all genetic backgrounds (i.e., single mutants, double mutants, single mutants with RNAi of the other in a specific tissue, triple mutants, etc) should be examined.

We appreciate this comment and agree such studies would be quite interesting. However, we feel that this would be beyond the scope of the current manuscript. Specifically, we feel that examination of insulin and Tor signaling activity in nine different genetic backgrounds would represent an entirely new manuscript and require the generation of dozens of additional genetic strains.

In all cases for which the authors make conclusions based on immunofluorescence microscopy, the data could be strengthened by quantification and/or showing more than one image for each condition to show the range observed.

We agree with this comment and have now quantified immunostaining data for pJNK, Upd3 and STAT.

Lines 357-359: The uniformity, or not, of image acquisition settings across groups in a given experiment needs to be mentioned.

This statement is now included in mounting and imaging sections of the methods.

Moderate concerns
Line 139-140: Perhaps the statement “…we find no evidence to support this hypothesis” should not be so definite since Ldh and Gpdh1 expression was not examine in all tissues; the figures do not show, and the text does not mention, expression in Malpighian tubules or muscle in each of the single mutants.

We have altered the text to read: “this hypothesis is not supported by our current set of observations.” (Line 140-141)

Line 151: Efficiency of Ldh RNAi-mediated knockdown is not mentioned. If this is shown in a previous manuscript, please provide references.

We previously demonstrated that the Ldh-RNAi line used in this study effectively reduces LDH protein levels (See Li et al. 2017), and have now noted this in the text (see lines 480-481).

Lines 181-182: Provide some rationale for focusing on upd3.

We have added the rationale (Lines 195-197).

Lines 208-209: Maybe reframe the setup of this section to be more direct, i.e., to provide support that impacts on metabolism are not the cause of the double mutant larval arrest. As written, the text leads the reader to expect to see assessment of many potential underlying causes.

We’ve made significant edits to the entire manuscript and hope this confusion is no longer an issue.

Lines 219-231: It would be interesting to assess if JAK/STAT signaling is altered in other tissues when Ldh has been knocked down specifically in one tissue in the Gpdh1 mutant background (like was done for Figure 2).

We agree that this would be an interesting experiment, but have decided to address this question in a future study, as any positive result would take significant effort to determine if such changes were due to Upd3, one of the other secrete factors, or changes in ecdysone synthesis and release.

Minor concerns
Line 130: missing the word “in” after “cells”

We have corrected that.

Error bars on all graphs: Since the color of the error bars is exactly the same as the symbol color, it is difficult to see the error bars. Perhaps change the color to black or a lighter shade of the symbol color and put the error bars on top.

We have changed the colors of the error bars in all Figures.

Figure 3: At first glance, it isn’t clear that the factors listed are DEGs in the double mutant. To make this clearer from the figure alone, perhaps put the text on top of a green box.

We have moved the DEGs within the figure.

**Reviewer #3:** In this study entitled “Glycolytic disruption restricts Drosophila melanogaster larval growth via the cytokine Upd3”, the authors describe a systemic signaling defect arising as a consequence of metabolic disruption in a specific tissue, primarily the muscle.
Loss of Gpdh1 and Ldh in the muscle leads to elevated Upd3 that drives developmental arrest by affecting the PG gland and dampened 20HE signaling. As a result, when the authors conduct Upd3 LOF in the Gpdh and Ldh double LOF condition they are able to restore the developmental arrest, thereby showing that the mediator of arrest is not a metabolic distress but rather a systemic signalling defect.

Overall, the study is interesting and implicates the importance of tissue specific metabolic state as a significant controller of organismal homeostasis.

Thank you for the kind comments!

I find the manuscript overall very important as it is in the realms of an area which is not thoroughly understood. The introduction is indeed very well written and positions the work very well. This raises the expectation when reading the manuscript, which however, then remains limited in terms of the core conceptual advancement that the study makes. Any understanding of how tissue specific metabolic states become deciding factors in coordinating organismal level development remains unanswered. As also pointed by the authors in the introduction, where this area of research is a relatively new, I suggest the authors to elaborate on this context as it is needed to strengthen the core problem posed in the study. Specifically:

1. Conceptual comments:
a. What is the relevance of the glycolytic state of the muscle with respect to the rest of the animal in homeostatic condition? What conditions influence this metabolic state is not described.

We agree that this relevance was lacking. We believe the key to the phenotype stems from the production of reductive stress in the double mutant muscle – these animals are unable to oxidize cytosolic NADH at a normal rate and are likely experiencing aspects of the metabolic stress present during hypoxia and ischemia-reprofusion injury. We have added a significant discussion of this in the discussion. See lines 365-382 as an example.

b. What is the lactate produced or G3P produced in normal homeostasis doing? Are they sensed by the PG to keep STAT activity in check allowing 20HE production and developmental continuity?

This is an interesting idea – that lactate, for example, might as a signaling molecule and loss of lactate in *Ldh* mutants promotes the larval arrest. We have added text to the discussion raising this possibility (Lines 448-456). In addition, we now clearly state our favored model in the discussion – that the key metabolic disruption caused by loss of Ldh and Gpdh1 is decreased capacity to oxidize NADH, which would inevitably place the animals in a state of reductive stress.

c. Does lactate supplementation in Ldh, Gpdh double mutant restore the growth delay? I understand the signaling downstream that the authors provide the data for when the tissue undergoes a disruption of metabolic homeostasis but in homeostasis, any understanding of the metabolites and what they contribute to remains unaddressed.

We’ve tried testing this possibility in the past via lactate supplementation, but excess dietary lactate proved quite detrimental to the development of Ldh mutants. We are uncertain as to why this is the case and there are many explanations, including the potential for induced lethal acidosis. Since deciphering the meaning of this phenomenon would require substantial effort, we will examine this possibility in a future study.

d. Finally, any physiological state that correlates with Ldh/gpdh lof condition is not mentioned. This will help the study and the readers with a context and a perspective that is currently missing. I strongly feel a physiological state recapitulating the Ldh/gpdh lof condition will tremendously help the current narrative.

We have added a substantial amount of new text discussing the importance of how Ldh and Gpdh1 maintains metabolic and redox homeostasis in our discussion section.

2. Experimental comments:
a. have the authors checked for other Upds? Even if negative result this will be important to know the specificity.

We intend to examine the other Upds in a future study. While *upd1* and *upd2* expression was unchanged in RNA-seq analysis of the double mutant, both genes could be significantly altered in individual tissues that would go unnoticed in our whole animal study. We have added text to the discussion explaining this possibility and the importance of examining them in future studies (Lines 420-437).

b. Have the authors driven Mef2>Gpdh1 RNAi in the Ldh mutant, the converse experiment, and do they recapitulate the same result?

We’ve attempted this reciprocal experiment using *Gpdh1* RNAi, however, we found that multiple TRiP lines failed to effectively reduce the expression of Gpdh1 protein, as determined by antibody staining. We’ve noted this in the text and included a supplemental figure (Figure S5) describing the problem.

c. How does the disruption in glycolytic state lead to JNK activation?

In short, we don’t know. Multiple studies have shown that JNK activation is often a stress response due to infection, tumor, wound or other processes. One interesting possibility is that JNK signaling is activated by reductive stress – a metabolic state that is poorly studied but plays an essential role in hypoxia and ischemia-reperfusion injuries (see Lines 335-349(. We are actively exploring this question – co-author Tennessen hypothesizes that the elevated reductive capacity of the cell alters disulfide bonds within key signaling molecules. Future proteomics approaches hold promise in answering this question.

d. Does driving JNK or Upd3 activation in the muscle, lead to the same phenotype as ldh/gpdh1 LOF?

This was an excellent suggestion! We conducted this experiment and found that muscle-specific Upd3 expression induced delayed development and also significantly disrupted pupal formation – 50% of the resulting pupae contained larval-like cuticle over the poster region. This data is now included as Figure 5.

e. A model will be good in the end that summarizes the story.

We now include a model figure (Figure 9) and have significantly expanded our discussion.

Overall, it is a relevant piece of work and I recommend the authors to explore the physiological inputs, as it will broaden the current scope of the work tremendous
